# Associations between smoking status and infertility: a cross-sectional analysis among USA women aged 18-45 years

**DOI:** 10.3389/fendo.2023.1140739

**Published:** 2023-04-19

**Authors:** Sijie He, Li Wan

**Affiliations:** Department of Pharmacy, Maternal and Child Health Hospital of Hubei Province, Tongji Medical College, Huazhong University of Science and Technology, Wuhan, China

**Keywords:** smoking status, infertility, NHANES, cross-sectional analysis, population-based study

## Abstract

**Background:**

Although many studies have proven the harmful effects of smoking on human health, the associations between smoking status and infertility are limited in large epidemiologic studies. We aimed to investigate the associations between smoking status and infertility among child-bearing women in the United States of America (USA).

**Methods:**

A total of 3,665 female participants (aged 18-45) from the National Health and Nutrition Examination Survey (NHANES) (2013-2018) were included in this analysis. All data were survey-weighted, and corresponding logistic regression models were performed to investigate the associations between smoking status and infertility.

**Results:**

In a fully adjusted model, the risk of infertility was found to be increased by 41.8% among current smokers compared to never smokers (95% CI: 1.044-1.926, *P*=0.025). In the subgroup analysis, the odds ratios (95% CI) of the risk of infertility for current smokers were 2.352 (1.018-5.435) in the unadjusted model for Mexican American, 3.675 (1.531-8.820) in the unadjusted model but 2.162 (0.946-4.942) in fully adjusted model for people aged 25-31, 2.201 (1.097-4.418) in the unadjusted model but 0.837 (0.435-1.612) in fully adjusted model for people aged 32-38.

**Conclusion:**

Current smokers was associated with a higher risk of infertility. The underlying mechanism of these correlations still needs more research. Our findings indicated that quitting smoking may serve as a simple index to reduce the risk of infertility.

## 1 Introduction

Infertility is a top public health concern which is defined as the failure to conceive within a year of unprotected sexual activity (1, 2). The United States’ Centers for Disease Control and Prevention (CDC) underlined that infertility was a serious public health concern with significant quality-of-life effects, such as psychological suffering, social stigma, financial strain, and marital discord (3). 15% of couples who are of childbearing age were struggling with infertility in the world (4). Although infertility is a widespread health problem, seldom are modifiable risk factors identified. Infertility has a complicated etiology that involves both male and female components, as well as a mixture of both. As of this writing, the CDC has designated infertility diagnosis and treatment as a national public health priority (5).

Cigarette smoking is a leading and preventable cause of morbidity and mortality worldwide (6–9). In the United States of America (USA), 34.1 million of individuals were reported to be smokers in 2019 (10). Smoking has so far been repeatedly shown to contribute to a wide range of human ailments, including reproductive abnormalities (11–13). About 4000 different chemicals, including alkaloids, heavy metals, and polycyclic aromatic hydrocarbons, all of which have reproductive toxicity are present in cigarette smoke (14, 15). Most research indicates that women who are current smokers and those who were exposed to parental smoking before conception had lower natural fertility (16).

By estimating an overall 60% increase in the probability of infertility, a meta-analysis highlighted a significant correlation between smoking and infertility (17). On the other hand, after controlling for relevant confounders, a prospective study was unable to find any discernible difference in fertility between smokers and non-smokers (18). In conclusion, there is conflicting evidence in the literature about the relationship between smoking and infertility. Other than that, the majority of earlier studies, however, used clinic-based samples, and only a few of them concentrated on sizable population-level samples. Considering the inconsistent and limited evidence on the associations between smoking status and infertility, based on a large national population-based representative survey, the objectives of this study were to evaluate the associations between smoking status and female infertility and determine which type of smoking status was linked with the highest infertility risk based on a population-based study. Age and race/ethnicity difference were further studied in the subgroup analysis because previous studies had demonstrated that they had an effect on the prevalence of infertility (19–22). The decision-making process by health authorities regarding programs for health promotion and intervention to avoid infertility in women of reproductive age may be aided by knowledge of the associations.

## 2 Methods

### 2.1 Data source and study population

NHANES, administered by the CDC and Prevention, is a nationally representative, cross-sectional survey conducted incessantly in 2-year cycles through questionnaire surveys, physical examinations, household interviews, and laboratory tests, designed to evaluate and assess the health and nutrition status of Americans. The included samples in this study have good representativeness because of the stratified multistage probability sampling approach used (23). The public can access all NHANES data at www.cdc.gov/nchs/nhanes/.

**Figure 1:**
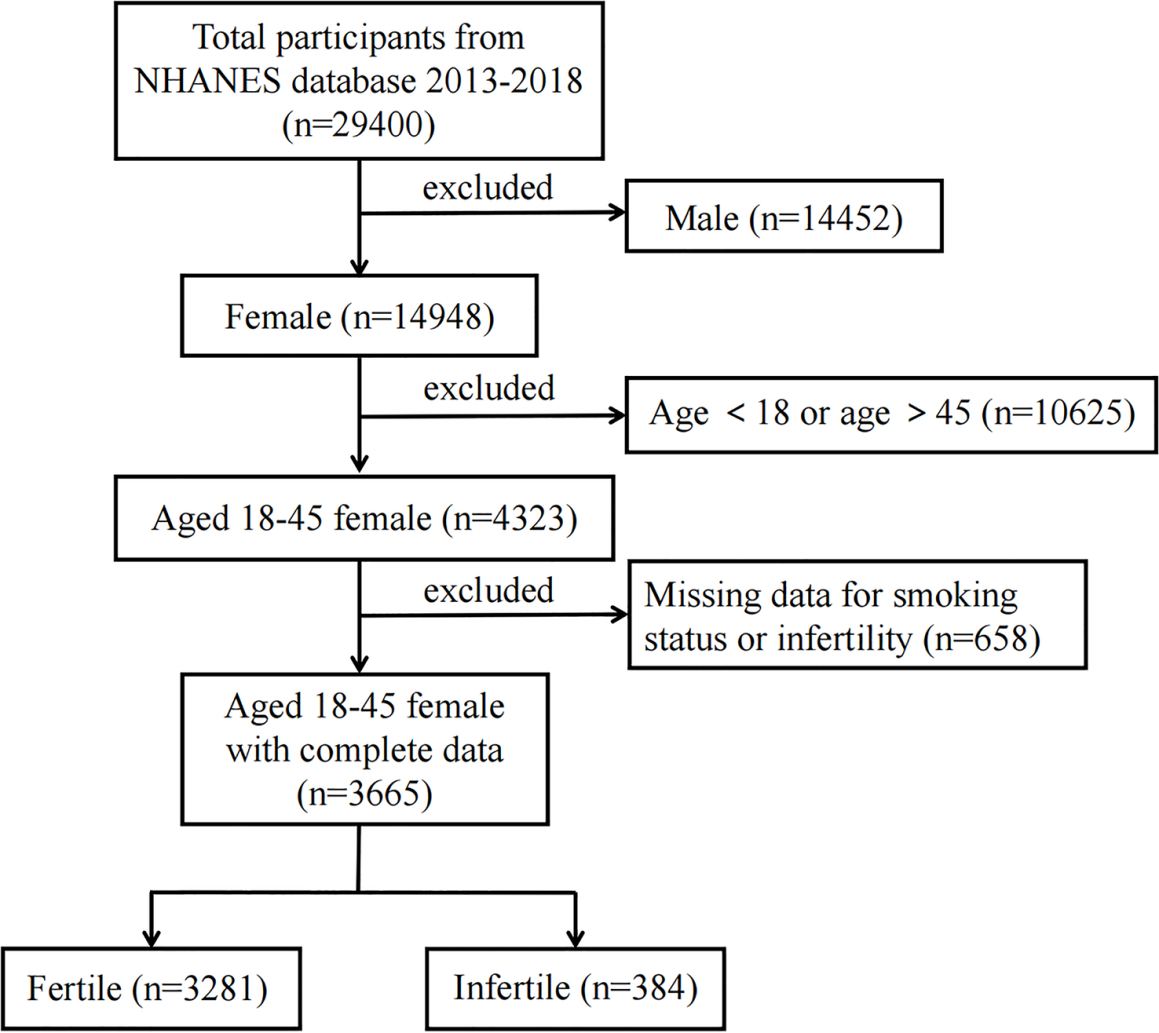
Flow chart of the study population. Describes how the sample of participants was composed. NHANES, National Health and Nutrition Examination Survey.

In the present study, NHANES data from 2013-2014, 2015-2016 and 2017-2018 were used. A total of 29,400 participants were incorporated at first; after the exclusion of males (n = 14,452), individuals aged <18 or >45 (n = 10,625) (24, 25), missing the smoking status or infertility data (n = 658), 3,665 participants were included in our final analysis (**Figure 1**). The National Center for Health Statistics Ethics Review Board approved human subjects for the conduction of NHANES, and all participants provided their written informed consent.

### 2.2 Study variables

In our analysis, the main exposure of interest is smoking status. Information on this exposure was obtained from relevant NHANES questionnaire items, which defined never smokers as having smoked fewer than 100 cigarettes in their lives, and former smokers as having smoked at least 100 cigarettes in their lives but not currently. These inquiries were made by trained interviewers using the Computer-Assisted Personal Interview (CAPI) technology at the mobile examination center (MEC). For these inquiries, both interpreters and proxy interviewers were accepted. Those who responded “Every day or certain days” to the question “Do you now smoke cigarettes?” were considered current smokers. Infertility is the key outcome of interest. These inquiries were made in the questionnaire on reproductive health using computer-assisted personal interviews conducted by qualified interviewers. Women who responded “yes” to the question “Have you ever attempted to become pregnant over a period of at least a year without becoming pregnant?” were labeled as experiencing infertility, with the answer “no” as the opposite category.

Our multivariable-adjusted models outlined relevant factors that might obscure the relationship between smoking status and infertility based on prior research (26). In our study, covariates including age (years), race/ethnicity (Mexican American/other Hispanic/non-Hispanic White/non-Hispanic Black/other races), education level (less than high school/high school/more than high school), marital status (married/living with partner, widowed/divorced/separated, never married) and ratio of family income to poverty (PIR), body mass index (BMI), diabetes, and physical activity were examined. According to Physical Activity Guidelines for Americans, 2nd edition of 75 min/week of vigorous or 150 min/week of moderate physical activity (27), participants were divided into three groups, including active, less active, and inactive. All detailed measurement procedures of the above variables are available on the NHANES website (www.cdc.gov/nchs/nhanes/).

### 2.3 Statistical analysis

For all statistical analyses, Stata (version 16.0), and EmpowerStats (version 2.0) were utilized with a determination of *P* < 0.05 for statistically significant. All estimates were computed using sample weights in accordance with National Center for Health Statistics’ analytical standards since NHANES seeks to create data that is representative of the civilian noninstitutionalized population in the USA. Continuous variables were characterized by mean ± SD if they were normally distributed and by median values and interquartile ranges otherwise. Percentages were used to characterize categorical variables which were compared by χ^2^ testing. To evaluate the relationship between smoking status and infertility, multivariable logistic regression was performed and the odds ratio (OR) values and 95% confidence intervals (95%CI) were calculated (28–32). Three models were built for the multivariate test. Model 1 had no variables adjusted. In Model 2, age and race/ethnicity were adjusted. Model 3 was adjusted for all covariates. Subgroup analysis stratified by age and race/ethnicity was carried out using stratified multivariate regression analysis.

## 3 Results

### 3.1 Baseline characteristics of participants

The weighted baseline characteristics of the included participants are displayed in **Table 1**. A total of 3,665 female participants were incorporated, including 384 with infertility and 3665 without infertility, with an average age of 31.438 ± 8.122 years. Infertility was significantly more prevalent among women who were older at the time of the survey (35.367 years vs. 30.924 years, *P* < 0.001), had higher BMI (≥30 kg/m^2^: 55.061% vs 36.871%, *P* < 0.001) and higher family income (PIR >1.85: 65.737% vs 57.792%, *P* = 0.008), and they tended to be never married (Married/Living with partner: 77.152% vs. 57.500%, *P <*0.001). In addition, they were more inclined to suffer from diabetes (7.955% vs 7.955%, *P <*0.001) and more intend to be current smokers (23.859% vs 18.139%, *P* = 0.001).

**Table 1:** Characteristics of the study population, National Health and Nutrition Examination Survey (NHANES) 2013–2018.

Characteristic	Total (n=3665)	No infertility (n=3281)	Infertility (n=384)	*P* value
**Age (years)**	31.438 ± 8.122	30.924 ± 8.107	35.367 ± 7.099	<0.001
**Race/Ethnicity (%)**				0.116
Mexican American	11.995	12.218	10.284	
Other Hispanic	7.908	8.075	6.631	
Non-Hispanic White	55.979	55.184	62.058	
Non-Hispanic Black	13.479	13.645	12.216	
Other race	10.639	10.878	8.810	
**Education (%)**				0.795
Less than high school	11.544	11.682	10.556	
High school	19.185	19.146	19.464	
More than high school	69.271	69.172	69.980	
**Marital status (%)**				<0.001
Married/Living with partner	29.766	32.279	11.832	
Widowed/Divorced/Separated	10.319	10.222	11.016	
Never Married	59.915	57.500	77.152	
**PIR (%)**				0.008
≤ 1.30	29.636	30.210	25.369	
1.30- ≤ 1.85	11.630	11.998	8.894	
> 1.85	58.734	57.792	65.737	
**BMI(%)**				<0.001
<25	36.806	38.036	27.382	
25≤-30	24.223	25.093	17.557	
≥30	38.971	36.871	55.061	
**Diabetes (%)**				<0.001
No	96.502	97.085	92.045	
Yes	3.498	2.915	7.955	
**Physical activity (%)**				0.698
Inactive	54.379	54.159	56.055	
Less active	7.342	7.440	6.598	
Active	38.279	38.401	37.347	
**Smoking status(%)**				0.001
Never smokers	69.427	70.439	61.686	
Former smokers	11.773	11.422	14.456	
Current smokers	18.800	18.139	23.859	

Mean ± SD for continuous variables: P value was calculated by one-way ANOVA; % for categorical variables: P value was calculated by χ^2^ test.

PIR, family income to poverty ratio; BMI, body mass index.

### 3.2 Associations between smoking status and infertility

Using binary logistic regression with single and multiple variables, we constructed three models to investigate relationships between smoking status and infertility. The pertinent effect size OR, 95%CI, and *P*-values are displayed in **Table 2**. There was a substantial correlation between smoking status and infertility in Models 1, 2, and 3, which was positive regardless of the kind of adjusted covariates. In the initial model (Model 1), the risk of infertility among current smokers increased by 54.9% than that among never smokers (OR=1.549; 95% CI: 1.189-2.017, *P*=0.001), 33.6% in Model 2 (OR: 1.336, 95% CI: 1.013-1.763, *P*=0.040) and 41.8% in Model 3 (OR: 1.418, 95% CI: 1.044-1.926, *P*=0.025).

**Table 2:** Association between smoking status and infertility.

	Model 1: OR (95%CI) *P*	Model 2: OR (95%CI) *P*	Model 3: OR (95%CI) *P*
Smoking status			
Never smokers	reference	reference	reference
Former smokers	1.629 (1.179, 2.252) 0.003	1.312 (0.939, 1.832) 0.112	1.152 (0.806, 1.646) 0.437
Current smokers	1.549 (1.189, 2.017) 0.001	1.336 (1.013, 1.763) 0.040	1.418 (1.044, 1.926) 0.025
*P* for trend	<0.001	<0.001	<0.001

Model 1 adjusted for none.

Model 2 adjusted for age and race/ethnicity.

Model 3 adjusted for age, race/ethnicity, education level, marital status, PIR, BMI, diabetes, and physical activity.

### 3.3 Subgroup analysis

Subgroup analysis revealed that the connection between smoking status and infertility was mostly present in Mexican Americans and participants aged 25 to 38 after controlling for variables. **Tables 3**, **4** provide comprehensive information on the subgroup analysis. For Mexican American, the association is similar in Model 2 (OR: 2.304, 95% CI: 0.971-5.470, *P*=0.058) and Model 3 (OR: 1.883, 95% CI: 0.829-4.278, *P*=0.13), but not in Model 1 (OR: 2.352, 95% CI: 1.018-5.435, *P*=0.045) (**Table 3**). For people aged 25-31, there is a significant positive association between smoking status and infertility in Model 1 (OR: 3.675, 95% CI: 1.531-8.820, *P*=0.004), Model 2 (OR: 2.501, 95% CI: 1.225-5.105, *P*=0.012) but not in Model 3 (OR: 2.162, 95% CI: 0.946-4.942, *P*=0.067). For people aged 32-38, there is a significant positive association in Model 1 (OR: 2.201, 95% CI: 1.097-4.418, *P*=0.026), but not in Model 2 (OR: 0.659, 95% CI: 0.363-1.195, *P*=0.169) and Model3 (OR: 0.837, 95% CI: 0.435-1.612, *P*=0.595) (**Table 4**).

**Table 3:** Association between smoking status and infertility stratified by race/ethnicity.

Race/Ethnicity (%)	Model 1 OR (95%CI) *P*	Model 2 OR (95%CI) *P*	Model 3 OR (95%CI) *P*
Mexican American			
Never smokers	reference	reference	reference
Former smokers	2.146 (0.860,5.356) 0.102	2.081 (0.824,5.254) 0.121	1.673 (0.579,4.831) 0.342
Current smokers	2.352 (1.018,5.435) 0.045	2.304 (0.971,5.470) 0.058	1.883 (0.829,4.278) 0.130
Other Hispanic			
Never smokers	reference	reference	reference
Former smokers	2.269 (0.813,6.338) 0.118	2.008 (0.699,5.768) 0.196	2.065 (0.673,6.342) 0.205
Current smokers	1.030 (0.316,3.362) 0.960	1.133 (0.343,3.748) 0.838	1.130 (0.284,4.496) 0.862
Non-Hispanic White			
Never smokers	reference	reference	reference
Former smokers	1.204 (0.690,2.100) 0.514	0.908 (0.503,1.639) 0.749	0.821 (0.445,1.513) 0.527
Current smokers	1.310 (0.837,2.049) 0.238	1.180 (0.743,1.874) 0.482	1.231 (0.723,2.098) 0.444
Non-Hispanic Black			
Never smokers	reference	reference	reference
Former smokers	1.405 (0.557,3.546) 0.471	1.239 (0.482,3.184) 0.656	1.112 (0.446,2.772) 0.820
Current smokers	1.726 (0.987,3.020) 0.056	1.608 (0.910,2.84) 0.1020	1.495 (0.786,2.843) 0.221
Other Race			
Never smokers	reference	reference	reference
Former smokers	1.652 (0.672,4.063) 0.274	1.523 (0.612,3.788) 0.365	1.305 (0.445,3.829) 0.627
Current smokers	1.696 (0.593,4.851) 0.324	1.787 (0.577,5.539) 0.314	1.921 (0.472,7.823) 0.362

Model 1 adjusted for none.

Model 2 adjusted for age and race/ethnicity.

Model 3 adjusted for age, race/ethnicity, education level, marital status, PIR, BMI, diabetes, and physical activity.

**Table 4:** Association between smoking status and infertility stratified by age.

Age	Model 1 OR (95%CI) *P*	Model 2 OR (95%CI) *P*	Model 3 OR (95%CI) *P*
Age (25-31)			
Never smokers	reference	reference	reference
Former smokers	2.587 (0.864,7.751) 0.089	1.620 (0.647,4.058) 0.303	1.225 (0.448,3.351) 0.693
Current smokers	3.675 (1.531,8.820) 0.004	2.501 (1.225,5.105) 0.012	2.162 (0.946,4.942) 0.067
Age (32-38)			
Never smokers	reference	reference	reference
Former smokers	1.523 (0.609,3.808) 0.368	1.404 (0.745,2.646) 0.293	1.511 (0.775,2.946) 0.226
Current smokers	2.201 (1.097,4.418) 0.026	0.659 (0.363,1.195) 0.169	0.837 (0.435,1.612) 0.595
Age (39-45)			
Never smokers	reference	reference	reference
Former smokers	1.488 (0.801,2.764) 0.208	0.636 (0.302,1.338) 0.233	0.633 (0.301,1.331) 0.228
Current smokers	0.711 (0.398,1.269) 0.248	1.266 (0.706,2.268) 0.429	1.619 (0.843,3.109) 0.148

Model 1 adjusted for none.

Model 2 adjusted for age and race/ethnicity.

Model 3 adjusted for age, race/ethnicity, education level, marital status, PIR, BMI, diabetes, and physical activity.

## 4 Discussion

In this cross-sectional study, which included 3,665 people, we found that current smokers had a higher risk of infertility. An examination of subgroups revealed that populations with Mexican American heritage and those between the ages of 25 and 38 shared this connection. Our findings imply that smoking status should be taken into account while treating infertile individuals in therapeutic settings.

Clinical investigations on the connection between smoking status and infertility in females are still limited and controversial. Three studies indicated a substantial link between smoking and infertility, with the risk being 1.85 (95% CI: 1.08-3.14) times greater for smokers than for non-smokers (33–35). The relevant literature from 1966 through late 1997 was found by a meta-analysis, which revealed an OR of 1.60 for infertility among female smokers compared to non-smokers across all research designs (23). Since the publication of this meta-analysis, more extensive population-based studies have shown that smoking has a detrimental effect on fecundity, regardless of other factors. The largest of these studies found that active smoking was linked to an increased failure to conceive within both the 6- and 12-month trial periods (36). However, this study divided smoking into active, passive, or both and the number of cigarettes smoked instead of different status and we are unclear about the difference between the relationship of past and current smoking and infertility. An Ontario, Canada, retrospective cohort study of farm couples found no difference in the risk of infertility between current smokers and non-smokers (37). Additionally, based on data from a North American internet-based preconception cohort study that enrolled participants from 2013 to 2018, a prospective analysis of cigarette smoking and fecundability found both female current smoking and previous smoking were related to slight declines in fecundity (38). Both of the above studies are consistent with the findings of Model 3 in our study, indicating that the relationship between smoking and infertility varies from current smoking status. In our study, the relationship between former smokers and infertility in **Table 2** was significant in Model 1 but not in Models 2 and 3 after adjusting for the covariate. The effect of former smokers on the outcome event infertility reflected not only the pure effect of exposure factor but also the effect of confounding factors. By constructing a multiple regression model in Model 2 and Model 3, i.e., “adjusting” for the effects of other confounding factors, the effect of the confounding factor was actually separated from the effect of the exposure factor. After eliminating the effect of the confounding factors, the spurious association between former smokers and the dependent variable disappeared, and there was no significant correlation between former smokers and infertility in Model 2 and Model 3. However, the population included in those studies did not distinguish between race and age. Thus, both age and race were the limitations of their study. In our study, the relationship between smoking status and infertility was found to be different across race and age groups by performing subgroup analysis.

Age and race/ethnicity have been proven in prior research to have an impact on the prevalence of infertility (19–22). Fecundity reportedly decreased for females in their late thirties and early forties. The likelihood of infertility rose from 10%-20% after age 35 to 45% in the early forties among women with previously confirmed fertility. Women who had never given birth were more likely to experience infertility at any age (19). Additionally, a study found that American Indians and Alaska Natives had a 1.30 times higher prevalence of decreased fecundity than white people (95% CI: 1.04 -1.62) (22). Thus, we conducted stratified analyses by age and race/ethnicity in the subgroup analysis.

Although the mechanisms underlying smoking and the risk of infertility have not been entirely understood, some evidence can support the negative association between them. Strong evidence suggested that smoking might impact natural female fertility by affecting several female reproductive function elements such as the ovary, oviduct, and uterus (39–41). In addition to clinical observational research in people, experiments on human tissues and cells as well as animal models have been used to study how smoking affects female reproductive function and fertility (42). But there is still debate over the outcomes. According to several research, smoking lowered the number of oocytes that may be obtained for assisted reproductive technology (ART) (43, 44). Those that are collected have a lower chance of becoming fertile, which lowers the quality of the resulting embryos (44, 45). However, when smokers are compared to age-matched controls, other studies have not discovered any differences in oocyte number, fertilization, embryo quality, clinical pregnancy, or birth rates (46, 47). The precise mechanism of the association between smoking status and infertility in our study remains unclear.

This study has a number of advantages. First, this study was based on data from NHANES, which are population-based sampling data collected across the country following a set procedure. The study samples were more representative since all analyses took into account the proper NHANES sampling weights. To make the results from the current study more trustworthy, the authors additionally made adjustments for confounding factors. However, it is impossible to disregard the study’s limitations. First, a clear causal association cannot be established by the authors because of the cross-sectional study methodology. Second, we were constrained in our secondary analysis due to our inability to gather fresh data. Therefore, there is a chance that unmeasured factors will cause residual confounding. For instance, because these data were not obtained, we were unable to control for the family history of infertility, a potentially significant confounder. To learn more about the harmful effects of smoking, it is crucial to investigate the relationship between smoking status and female infertility. More studies are still required to produce definitive pieces of data.

## 5 Conclusion

This study demonstrated that current smoking was associated with elevated infertility risk. In subgroup analyses, the associations of smoking status with infertility were only found in women aged 25-38 and in Mexican Americans. Further studies are still needed to validate our findings.

## Data availability statement

Publicly available datasets were analyzed in this study. This data can be found here: https://www.cdc.gov/nchs/nhanes/index.htm.

## Author contributions

SH designed the research, analyzed the data, and wrote the paper. LW assisted in manuscript preparation. All authors contributed to the article and approved the submitted version.
